# Supplementary material for: SyNDI: synchronous network data integration framework
Source: BMC Bioinformatics. 2018 Nov 6;19:403. doi: 10.1186/s12859-018-2426-5 (PMC6219086; doi:10.1186/s12859-018-2426-5)
Supplement: Supplementary file 5 — Files of the example presented in Additional file 4. This additional file is a zip package that contains all files used in Additional file 4. Detailed descriptions of each of these files are presented in a README file included in this zip package. (GZ 30411 kb) [file 12859_2018_2426_MOESM5_ESM.gz › SyNDITools/Meme2Fimo/meme_4.9.0_4.tar.gz!/meme_4.9.0/etc/meme.doc]

MEME — Multiple EM for Motif Elicitation

USAGE:

     meme <dataset> [optional arguments]

<dataset>	file containing sequences in FASTA format

[-h]	print this message

[-o <output dir>]	name of directory for output files will not

replace existing directory

[-oc <output dir>]	name of directory for output files will

replace existing directory

[-text]	output in text format (default is HTML)

[-dna]	sequences use DNA alphabet

[-protein]	sequences use protein alphabet

[-mod oops|zoops|anr]	distribution of motifs

[-nmotifs <nmotifs>]	maximum number of motifs to find

[-evt <ev>]	stop if motif E-value greater than <evt>

[-nsites <sites>]	number of sites for each motif

[-minsites <minsites>] minimum number of sites for each motif

[-maxsites <maxsites>] maximum number of sites for each motif

[-wnsites <wnsites>]	weight on expected number of sites

[-w <w>]	motif width

[-minw <minw>]	minumum motif width

[-maxw <maxw>]	maximum motif width

[-nomatrim]	do not adjust motif width using multiple

alignments

[-wg <wg>]	gap opening cost for multiple alignments

[-ws <ws>]	gap extension cost for multiple alignments

[-noendgaps]	do not count end gaps in multiple alignments

[-bfile <bfile>]	name of background Markov model file

[-revcomp]	allow sites on + or - DNA strands

[-pal]	force palindromes (requires -dna)

[-maxiter <maxiter>]	maximum EM iterations to run

[-distance <distance>] EM convergence criterion

[-psp <pspfile>]	name of positional priors file

[-prior dirichlet|dmix| type of prior to use

  mega|megap|addone]

[-b <b>]	strength of the prior

[-plib <plib>]	name of Dirichlet prior file

[-spfuzz <spfuzz>]	fuzziness of sequence to theta mapping

[-spmap uni|pam]	starting point seq to theta mapping type

[-cons <cons>]	consensus sequence to start EM from

[-heapsize <hs>]	size of heaps for widths where substring

search occurs

[-x_branch]	perform x-branching

[-w_branch]	perform width branching

[-bfactor <bf>]	branching factor for branching search

[-maxsize <maxsize>]	maximum dataset size in characters

[-nostatus]	do not print progress reports to terminal

[-p <np>]	use parallel version with <np> processors

[-time <t>]	quit before <t> CPU seconds consumed

[-sf <sf>]	print <sf> as name of sequence file

[-V]	verbose mode

MEME is a tool for discovering motifs in a group of related DNA or protein

sequences.

A motif is a sequence pattern that occurs repeatedly in a group of related

protein or DNA sequences. MEME represents motifs as position-dependent

letter-probability matrices which describe the probability of each

possible letter at each position in the pattern. Individual MEME motifs do

not contain gaps. Patterns with variable-length gaps are split by MEME

into two or more separate motifs.

MEME takes as input a group of DNA or protein sequences (the training set)

and outputs as many motifs as requested. MEME uses statistical modeling

techniques to automatically choose the best width, number of occurrences,

and description for each motif.

MEME outputs its results primarily as a hypertext (HTML) document named

meme.html. This is placed in a directory named meme_out/". You can select

for the directory to have a different name. MEME also outputs

machine-readable (XML) a plain-text versions of its output, named meme.xml

and meme.txt, respectively. These are placed in the same output directory

as meme.html.

The MEME results consist of:

* The version of MEME and the date it was released.

* The reference to cite if you use MEME in your research.

* A description of the sequences you submitted (the "training set")

showing the name, "weight" and length of each sequence.

* The command line summary detailing the parameters with which you ran

MEME.

* Information on each of the motifs MEME discovered, including:

 1. A summary line showing the width, number of occurrences, log

likelihood ratio and statistical significance of the motif.

 2. A sequence "LOGO" illustrating the motif, along with links to

publication-ready versions of the LOGO (postscript and PNG

formats).

 3. The occurrences of the motif sorted by p-value and aligned with

each other.

 4. Block diagrams of the occurrences of the motif within each

sequence in the training set.

 5. The motif in BLOCKS format: buttons for viewing the motif in

various formats and for submitting it to the BLOCKS multiple

alignment processor.

 6. A position-specific scoring matrix (PSSM) for use in scanning

sequence databases and a button for submitting the motif to the

MAST scanning program.

 7. The position specific probability matrix (PSPM) describing the

motif and a button for comparing the motif to known motifs.

 8. A regular expression describing the motif.

* A summary of motifs showing an optimized (non-overlapping) tiling of

all of the motifs onto each of the sequences in the training set.

* The reason why MEME stopped and the name of the CPU on which it ran.

* This explanation of how to interpret MEME results.

REQUIRED ARGUMENTS:

* <dataset> The name of the file containing the training set sequences.

If <dataset> is the word stdin, MEME reads from standard input.

The sequences in the dataset should be in Pearson/FASTA format. For

example:

>ICYA_MANSE INSECTICYANIN A FORM (BLUE BILIPROTEIN)

GDIFYPGYCPDVKPVNDFDLSAFAGAWHEIAK

LPLENENQGKCTIAEYKYDGKKASVYNSFVSNGVKEYMEGDLEIAPDA

>LACB_BOVIN BETA-LACTOGLOBULIN PRECURSOR (BETA-LG)

MKCLLLALALTCGAQALIVTQTMKGLDI

QKVAGTWYSLAMAASDISLLDAQSAPLRVYVEELKPTPEGDLEILLQKW

Sequences start with a header line followed by sequence lines. A

header line has the character ">" in position one, followed by an

unique name without any spaces, followed by (optional) descriptive

text. After the header line come the actual sequence lines. Spaces and

blank lines are ignored. Sequences may be in capital or lowercase or

both.

MEME uses the first word in the header line of each sequence,

truncated to 24 characters if necessary, as the name of the sequence.

This name must be unique. Sequences with duplicate names will be

ignored. (The first word in the title line is everything following the

">" up to the first blank.)

Sequence weights may be specified in the dataset file by special

header lines where the unique name is "WEIGHTS" (all caps) and the

descriptive text is a list of sequence weights. Sequence weights are

numbers in the range 0 < w <= 1. All weights are assigned in order to

the sequences in the file. If there are more sequences than weights,

the remainder are given weight one. Weights must be greater than zero

and less than or equal to one. Weights may be specified by more than

one "WEIGHT" entry which may appear anywhere in the file. When weights

are used, sequences will contribute to motifs in proportion to their

weights. Here is an example for a file of three sequences where the

first two sequences are very similar and it is desired to down-weight

them:

>WEIGHTS 0.5 .5 1.0

>seq1

GDIFYPGYCPDVKPVNDFDLSAFAGAWHEIAK

>seq2

GDMFCPGYCPDVKPVGDFDLSAFAGAWHELAK

>seq3

QKVAGTWYSLAMAASDISLLDAQSAPLRVYVEELKPTPEGDLEILLQKW

OPTIONAL ARGUMENTS:

MEME has a large number of optional inputs that can be used to fine-tune

its behavior. To make these easier to understand they are divided into the

following categories:

* OUTPUT DESTINATION — control where MEME places its output

* ALPHABET — control the alphabet for the motifs (patterns) that MEME

will search for

* DISTRIBUTION — control how MEME assumes the occurrences of the motifs

are distributed throughout the training set sequences

* SEARCH — control how MEME searches for motifs

* SYSTEM — the -p <np> argument causes a version of MEME compiled for a

parallel CPU architecture to be run. (By placing <np> in quotes you

may pass installation specific switches to the 'mpirun' command. The

number of processors to run on must be the first argument following

-p).

In what follows, <n> is an integer, <a> is a decimal number, and <string>

is a string of characters.

OUTPUT DESTINATION

By default MEME writes its results to a directory named meme_out/, which

is created if it doesn't exist. The main results include file is

meme.html. You can specify that a different directory be created or used

for results. You can also specify that MEME create only a text file.

* -o <output dir> — Name of directory for output files; will not replace

existing directory.

* -oc <output dir> — Name of directory for output files; will replace

existing directory.

* -text — Output in text format only to standard output.

ALPHABET

MEME accepts either DNA or protein sequences, but not both in the same

run. By default, sequences are assumed to be protein. The sequences must

be in FASTA format.

DNA sequences must contain only the letters "ACGT", plus the ambiguous

letters "BDHKMNRSUVWY*-". Protein sequences must contain only the letters

"ACDEFGHIKLMNPQRSTVWY", plus the ambiguous letters "BUXZ*-".

MEME converts all ambiguous letters to "X", which is treated as "unknown".

* -dna — Assume sequences are DNA; default: protein sequences.

* -protein — Assume sequences are protein.

DISTRIBUTION

If you know how occurrences of motifs are distributed in the training set

sequences, you can specify it with the following optional switches. The

default distribution of motif occurrences is assumed to be zero or one

occurrence of per sequence.

If you know how occurrences of motifs are distributed in the training set

* -mod <string> — The type of distribution to assume.

* oops — One Occurrence Per Sequence MEME assumes that each

sequence in the dataset contains exactly one occurrence of each

motif. This option is the fastest and most sensitive but the

motifs returned by MEME may be "blurry" if any of the sequences

is missing them.

* zoops — Zero or One Occurrence Per Sequence MEME assumes that

each sequence may contain at most one occurrence of each motif.

This option is useful when you suspect that some motifs may be

missing from some of the sequences. In that case, the motifs

found will be more accurate than using the first option. This

option takes more computer time than the first option (about

twice as much) and is slightly less sensitive to weak motifs

present in all of the sequences.

* anr — Any Number of Repetitions MEME assumes each sequence may

contain any number of non-overlapping occurrences of each motif.

This option is useful when you suspect that motifs repeat

multiple times within a single sequence. In that case, the motifs

found will be much more accurate than using one of the other

options. This option can also be used to discover repeats within

a single sequence. This option takes the much more computer time

than the first option (about ten times as much) and is somewhat

less sensitive to weak motifs which do not repeat within a single

sequence than the other two options.

SEARCH

 1. OBJECTIVE FUNCTION

MEME uses an objective function on motifs to select the "best" motif.

The objective function is based on the statistical significance of the

log likelihood ratio (LLR) of the occurrences of the motif. The

E-value of the motif is an estimate of the number of motifs (with the

same width and number of occurrences) that would have equal or higher

log likelihood ratio if the training set sequences had been generated

randomly according to the (0-order portion of the) background model.

MEME searches for the motif with the smallest E-value. It searches

over different motif widths, numbers of occurrences, and positions in

the training set for the motif occurrences. The user may limit the

range of motif widths and number of occurrences that MEME tries using

the switches described below. In addition, MEME trims the motif (using

a dynamic programming multiple alignment) to eliminate any positions

where there is a gap in any of the occurrences.

The log likelihood ratio of a motif is llr = log (Pr(sites | motif) /

Pr(sites | back)) and is a measure of how different the sites are from

the background model. Pr(sites | motif) is the probability of the

occurrences given the a model consisting of the position-specific

probability matrix (PSPM) of the motif. (The PSPM is output by MEME).

Pr(sites | back) is the probability of the occurrences given the

background model. The background model is an n-order Markov model. By

default, it is a 0-order model consisting of the frequencies of the

letters in the training set. A different 0-order Markov model or

higher order Markov models can be specified to MEME using the -bfile

option described below.

The E-value reported by MEME is actually an approximation of the

E-value of the log likelihood ratio. (An approximation is used because

it is far more efficient to compute.) The approximation is based on

the fact that the log likelihood ratio of a motif is the sum of the

log likelihood ratios of each column of the motif. Instead of

computing the statistical significance of this sum (its p-value), MEME

computes the p-value of each column and then computes the significance

of their product. Although not identical to the significance of the

log likelihood ratio, this easier to compute objective function works

very similarly in practice.

The motif significance is reported as the E-value of the motif. The

statistical significance of a motif is computed based on:

 1. the log likelihood ratio,

 2. the width of the motif,

 3. the number of occurrences,

 4. the 0-order portion of the background model,

 5. the size of the training set, and

 6. the type of model (oops, zoops, or anr, which determines the

number of possible different motifs of the given width and number

of occurrences).

MEME searches for motifs by performing Expectation Maximization (EM)

on a motif model of a fixed width and using an initial estimate of the

number of sites. It then sorts the possible sites according to their

probability according to EM. MEME then and calculates the E-values of

the first n sites in the sorted list for different values of n. This

procedure (first EM, followed by computing E-values for different

numbers of sites) is repeated with different widths and different

initial estimates of the number of sites. MEME outputs the motif with

the lowest E-value.

 2. NUMBER OF MOTIFS

* -nmotifs <n> — The number of *different* motifs to search for.

MEME will search for and output <n> motifs. Default: 1

* -evt <p> — Quit looking for motifs if E-value exceeds <p>.

Default: infinite (so by default MEME never quits before -nmotifs

<n> have been found.)

 3. NUMBER OF MOTIF OCCURRENCES

* -nsites <n>

-minsites <n>

-maxsites <n>

The (expected) number of occurrences of each motif. If -nsites is

given, only that number of occurrences is tried. Otherwise,

numbers of occurrences between -minsites and -maxsites are tried

as initial guesses for the number of motif occurrences. These

switches are ignored if mod = oops.

Defaults:

-minsites : 2

-maxsites :

zoops : number of sequences

anr	: MIN(5*(number of sequences), 50)

* -wnsites <n> — The weight on the prior on nsites. This controls

how strong the bias towards motifs with exactly nsites sites (or

between minsites and maxsites sites) is. It is a number in the

range [0..1). The larger it is, the stronger the bias towards

motifs with exactly nsites occurrences is.

Default: 0.8

 4. MOTIF WIDTH

* -w <n>

-minw <n>

-maxw <n>

The width of the motif(s) to search for. If -w is given, only

that width is tried. Otherwise, widths between -minw and -maxw

are tried.

Default: -minw 8, -maxw 50 (defined in user.h)

Note: If <n> is less than the length of the shortest sequence in

the dataset, <n> is reset by MEME to that value.

* -nomatrim

-wg <a>

-ws <a>

-noendgaps

These switches control trimming (shortening) of motifs using the

multiple alignment method. Specifying -nomatrim causes MEME to

skip this and causes the other switches to be ignored. MEME finds

the best motif found and then trims (shortens) it using the

multiple alignment method (described below). The number of

occurrences is then adjusted to maximize the motif E-value, and

then the motif width is further shortened to optimize the

E-value.

The multiple alignment method performs a separate pairwise

alignment of the site with the highest probability and each other

possible site. (The alignment includes width/2 positions on

either side of the sites.) The pairwise alignment is controlled

by the switches:

-wg <a> (gap cost; default: 11),

-ws <a> (space cost; default 1), and,

-noendgaps (do not penalize endgaps; default: penalize endgaps).

The pairwise alignments are then combined and the method

determines the widest section of the motif with no insertions or

deletions. If this alignment is shorter than <minw>, it tries to

find an alignment allowing up to one insertion/deletion per motif

column. This continues (allowing up to 2, 3 ...

insertions/deletions per motif column) until an alignment of

width at least <minw> is found.

 5. BACKGROUND MODEL

* -bfile <bfile> — The name of the file containing the background

model for sequences. The background model is the model of random

sequences used by MEME. The background model is used by MEME

 1. during EM as the "null model",

 2. for calculating the log likelihood ratio of a motif,

 3. for calculating the significance (E-value) of a motif, and,

 4. for creating the position-specific scoring matrix (log-odds

matrix).

By default, the background model is a 0-order Markov model based on

the letter frequencies in the training set.

Markov models of any order can be specified in <bfile> by listing

frequencies of all possible tuples of length up to order+1.

Note that MEME uses only the 0-order portion (single letter

frequencies) of the background model for purposes 3) and 4), but uses

the full-order model for purposes 1) and 2), above.

Example: To specify a 1-order Markov background model for DNA, <bfile>

might contain the following lines. Note that optional comment lines

are marked by "#" and are ignored by MEME.

\# tuple	frequency_non_coding

a	0.324

c	0.176

g	0.176

t	0.324

\# tuple	frequency_non_coding

aa	0.119

ac	0.052

ag	0.056

at	0.097

ca	0.058

cc	0.033

cg	0.028

ct	0.056

ga	0.056

gc	0.035

gg	0.033

gt	0.052

ta	0.091

tc	0.056

tg	0.058

tt	0.119

Sample -bfile files are given in directory tests: tests/nt.freq (DNA),

and tests/na.freq (amino acid).

 6. POSITION-SPECIFIC PRIORS

* -psp <pspfile> — position-specific prior (PSP)

These priors allow the user to bias the search for motifs. They

give a position-specific prior distribution on the location of

motif sites in sequence(s) in the input dataset. The MEME PSP

format used in the pspfile includes the name of the sequence for

which a prior distribution corresponds. Sequences not named in

the pspfile are given uniform prior distributions on site

locations by MEME.

A PSP must be created for a specific width of motif, w. This

width must be specified for each entry in the pspfile, and must

be the same for all entries. If MEME varies the motif width

during computation, MEME renormalises the PSP for each sequence.

The pspfile should be in MEME PSP format, which is similar to

FASTA format. For example:

>ICYA_MANSE 4

0.075922 0.070764 0.082380 0.030292 0.025101 0.043139 0.032963

0.086047 0.057445 0.000000 0.000000 0.000000

>LACB_BOVIN 4

0.107099 0.099822 0.116208 0.042731 0.035408 0.060854 0.046499

0.000000 0.000000 0.000000

Each entry should start with a header line consisting of a

sequence name followed by the width, w, of the PSP prior. The

sequence name must the name of a sequence in the FASTA file input

to MEME. Any other text on the header line after the name and w

is ignored by MEME. The following lines contain one number for

each position in the identically-named named FASTA sequence,

where the number gives the prior probability of a motif site at

that position in the sequence (or in the reverse complement if

-revcomp is specified). The last w-1 numbers for each entry

should be 0 (shown in blue in the example), since a motif of that

width cannot start in those positions. All numbers for an entry

must be in the range [0,1], and must sum to a number no greater

than 1. If they sum to less than 1 and -mod oops is specified,

MEME will rescale the numbers so that they sum to 1.

For more detail on generation of PSP data, see documentation of

our simple tool [1]psp-gen.

 7. DNA PALINDROMES AND STRANDS

* -revcomp — motifs occurrences may be on the given DNA strand or

on its reverse complement.

Default: look for DNA motifs only on the strand given in the

training set.

* -pal — Choosing -pal causes MEME to look for palindromes in DNA

datasets.

MEME averages the letter frequencies in corresponding columns of the

motif (PSPM) together. For instance, if the width of the motif is 10,

columns 1 and 10, 2 and 9, 3 and 8, etc., are averaged together. The

averaging combines the frequency of A in one column with T in the

other, and the frequency of C in one column with G in the other. If

neither option is not chosen, MEME does not search for DNA

palindromes.

 8. EM ALGORITHM

* -maxiter <n> — The number of iterations of EM to run from any

starting point. EM is run for <n> iterations or until convergence

(see -distance, below) from each starting point. Default: 50

* -distance <a> — The convergence criterion. MEME stops iterating

EM when the change in the motif frequency matrix is less than <a>

(Change is the euclidean distance between two successive

frequency matrices.) Default: 0.001

* -prior <string> — The prior distribution on the model parameters:

* dirichlet — simple Dirichlet prior. This is the default for

-dna. It is based on the non-redundant database letter

frequencies.

* dmix — mixture of Dirichlets prior. This is the default for

-protein.

* mega — extremely low variance dmix; variance is scaled

inversely with the size of the dataset.

* megap — mega for all but last iteration of EM; dmix on last

iteration.

* addone — add +1 to each observed count

* -b <a> — The strength of the prior on model parameters: <a> = 0

means use intrinsic strength of prior for prior = dmix.

Defaults:

* 0.01 — if prior = dirichlet

* 0 — if prior = dmix

* -plib <string> — The name of the file containing the Dirichlet

prior in the format of file prior30.plib.

 9. SELECTING STARTS FOR EM

The default is for MEME to search the dataset for good starts for EM.

How the starting points are derived from the dataset is specified by

the following switches.

The default type of mapping MEME uses is:

* -spmap uni — for -dna

* -spmap pam — for -protein

* -spfuzz <a> — The fuzziness of the mapping. Possible values are

greater than 0. Meaning depends on -spmap, see below.

* -spmap <string> — The type of mapping function to use.

* uni — Use add-<a> prior when converting a substring to an

estimate of theta.

Default -spfuzz <a> 0.5

* pam — Use columns of PAM <a> matrix when converting a

substring to an estimate of theta.

Default -spfuzz <a> 120 (PAM 120)

Other types of starting points can be specified using the

following switches.

* -cons <string> — Override the sampling of starting points and

just use a starting point derived from <string>. This is useful

when an actual occurrence of a motif is known and can be used as

the starting point for finding the motif.

10. BRANCHING SEARCH ON EM STARTS

The search for good EM starting points can be improved by using

branching search.

Branching search begins with a fixed-sized heap of best EM starts

identified during the search of subsequences from the dataset. These

starts are also called "seeds". The fixed-sized heap of seeds is used

as the "branch_heap" during the first iteration of branching search.

For each iteration of branching search, all seeds in the current

branch_heap are considered. All seeds in the ball within hamming

distance 1 of a given seed are evaluated and added to a new heap. The

ball of new seeds is generated by mutating each character of the

initial seed to each alternative character in the alphabet.

After the ball for every branch_heap seed has been evaluated, the

seeds in the resulting new heap are added to the heap of best EM

starts. The new heap is then used as the branch_heap for the next

iteration of branching search.

By default MEME does not perform x-branching.

* -x_branch — Perform x-branching.

* -bfactor <bf> — The number of iterations of branching search. The

default number of branching iterations is three.

* -heapsize <hs> — The maximum size of the heaps used during

branching search. The default heap size is 64.

EXAMPLES:

The following examples use data files provided in this release of MEME.

MEME writes its output to standard output, so you will want to redirect it

to a file in order for use with MAST.

 1. A simple DNA example:

meme crp0.s -dna -mod oops -pal

MEME looks for a single motif in the file crp0.s which contains DNA

sequences in FASTA format. The OOPS model is used so MEME assumes that

every sequence contains exactly one occurrence of the motif. The

palindrome switch is given so the motif model (PSPM) is converted into

a palindrome by combining corresponding frequency columns. MEME

automatically chooses the best width for the motif in this example

since no width was specified.

 2. Searching for motifs on both DNA strands:

meme crp0.s -dna -mod oops -revcomp

This is like the previous example except that the -revcomp switch

tells MEME to consider both DNA strands, and the -pal switch is absent

so the palindrome conversion is omitted. When DNA uses both DNA

strands, motif occurrences on the two strands may not overlap. That

is, any position in the sequence given in the training set may be

contained in an occurrence of a motif on the positive strand or the

negative strand, but not both.

 3. A fast DNA example:

meme crp0.s -dna -mod oops -revcomp -w 20

This example differs from example 1) in that MEME is told to only

consider motifs of width 20. This causes MEME to execute about 10

times faster. The -w switch can also be used with protein datasets if

the width of the motifs is known in advance.

 4. Using a higher-order background model:

meme INO_up800.s -dna -mod anr -revcomp -bfile yeast.nc.6.freq

In this example we use -mod anr and -bfile yeast.nc.6.freq. This

specifies that

a) the motif may have any number of occurrences in each sequence, and,

b) the Markov model specified in yeast.nc.6.freq is used as the

background model. This file contains a fifth-order Markov model for

the non-coding regions in the yeast genome.

Using a higher order background model can often result in more

sensitive detection of motifs. This is because the background model

more accurately models non-motif sequence, allowing MEME to

discriminate against it and find the true motifs.

 5. A simple protein example:

meme lipocalin.s -mod oops -maxw 20 -nmotifs 2

The -dna switch is absent, so MEME assumes the file lipocalin.s

contains protein sequences. MEME searches for two motifs each of width

less than or equal to 20. (Specifying -maxw 20 makes MEME run faster

since it does not have to consider motifs longer than 20.) Each motif

is assumed to occur in each of the sequences because the OOPS model is

specified.

 6. Another simple protein example:

meme farntrans5.s -mod anr -maxw 40 -maxsites 50

MEME searches for a motif of width up to 40 with up to 50 occurrences

in the entire training set. The ANR sequence model is specified, which

allows each motif to have any number of occurrences in each sequence.

This dataset contains motifs with multiple repeats of motifs in each

sequence. This example is fairly time consuming due to the fact that

the time required to initial the motif probability tables is

proportional to <maxw> times <maxsites>. By default, MEME only looks

for motifs up to 29 letters wide with a maximum total of number of

occurrences equal to twice the number of sequences or 30, whichever is

less.

 7. A much faster protein example:

meme farntrans5.s -mod anr -w 10 -maxsites 30 -nmotifs 3

This time MEME is constrained to search for three motifs of width

exactly ten. The effect is to break up the long motif found in the

previous example. The -w switch forces motifs to be *exactly* ten

letters wide. This example is much faster because, since only one

width is considered, the time to build the motif probability tables is

only proportional to <maxsites>.

 8. Splitting the sites into three:

meme farntrans5.s -mod anr -maxw 12 -nsites 24 -nmotifs 3

This forces each motif to have 24 occurrences, exactly, and be up to

12 letters wide.

 9. A larger protein example with E-value cutoff:

meme adh.s -mod zoops -nmotifs 20 -evt 0.01

In this example, MEME looks for up to 20 motifs, but stops when a

motif is found with E-value greater than 0.01. Motifs with large

E-values are likely to be statistical artifacts rather than

biologically significant.

References

Visible links

1. file:///home/james/dist/doc/psp-gen.html
